# Physiologic Consequences of Caveolin-1 Ablation in Conventional Outflow Endothelia

**DOI:** 10.1167/iovs.61.11.32

**Published:** 2020-09-17

**Authors:** Michael L. De Ieso, Jami M. Gurley, Mark E. McClellan, Xiaowu Gu, Iris Navarro, Guorong Li, Maria Gomez-Caraballo, Eric Enyong, W. Daniel Stamer, Michael H. Elliott

**Affiliations:** 1 Department of Ophthalmology, Duke Eye Center, Duke University, Durham, North Carolina, United States; 2 Department of Ophthalmology, Dean McGee Eye Institute University of Oklahoma Health Sciences Center, Oklahoma City, Oklahoma, United States; 3 Department of Physiology, University of Oklahoma Health Sciences Center, Oklahoma City, Oklahoma, United States

**Keywords:** caveolae, caveolin-1, glaucoma, intraocular pressure, eNOS, conventional outflow, endothelium, Schlemm's canal

## Abstract

**Purpose:**

Polymorphisms at the caveolin-1/2 locus are associated with glaucoma and IOP risk and deletion of caveolin-1 (Cav1) in mice elevates IOP and reduces outflow facility. However, the specific location/cell type responsible for Cav1-dependent regulation of IOP is unclear. We hypothesized that endothelial Cav1 in the conventional outflow (CO) pathway regulate IOP via endothelial nitric oxide synthase (eNOS) signaling.

**Methods:**

We created a mouse with targeted deletion of Cav1 in endothelial cells (Cav1^ΔEC^) and evaluated IOP, outflow facility, outflow pathway distal vascular morphology, eNOS phosphorylation, and tyrosine nitration of iridocorneal angle tissues by Western blotting.

**Results:**

Endothelial deletion of Cav1 resulted in significantly elevated IOP versus wild-type mice but not a concomitant decrease in outflow facility. Endothelial Cav1 deficiency did not alter the trabecular meshwork or Schlemm's canal morphology, suggesting that the effects observed were not due to developmental deformities. Endothelial Cav1 deletion resulted in eNOS hyperactivity, modestly increased protein nitration, and significant enlargement of the drainage vessels distal to Schlemm's canal. L-Nitro-arginine methyl ester treatment reduced outflow in Cav1^ΔEC^ but not wild-type mice and had no effect on the size of drainage vessels. Endothelin-1 treatment decrease the outflow and drainage vessel size in both wild-type and Cav1^ΔEC^ mice.

**Conclusions:**

Our results suggest that hyperactive eNOS signaling in the CO pathway of both Cav1^ΔEC^ and global Cav1 knockout mice results in chronic dilation of distal CO vessels and protein nitration, but that Cav1 expression in the trabecular meshwork is sufficient to rescue CO defects reported in global Cav1 knockout mice.

Elevated IOP or ocular hypertension is the primary risk factor for glaucoma,^1^ a leading cause of blindness worldwide.^2^ The reason for ocular hypertension is dysregulated removal of aqueous humor from the eye by the conventional outflow (CO) pathway. Fortunately, glaucoma is treatable with first-line topical medications that lower IOP by reducing aqueous humor secretion^3^^,^^4^ or augment outflow via the unconventional (secondary) outflow pathway.^5^^,^^6^ Neither strategy, however, targets disease in the CO pathway. Recently, new medications have been developed that increase CO, and are additive with current first-line treatments.^7^^,^^8^ Although these combination therapies provide a major improvement for glaucoma patients, there still is a clinical need for targeted CO treatments. Thus, research has intensified in order to understand the cellular and molecular mechanisms that regulate CO resistance.^8^

The CO pathway consists of three parts: the trabecular meshwork (TM), which is a porous connective tissue, the Schlemm's canal (SC), which is a specialized drainage vessel with dual blood vascular and lymphatic characteristics; and distal vessels that include collector channels, aqueous veins, and intrascleral venous plexus.^9^ Vasomotion in distal venous vessels creates some outflow resistance; however, the majority of resistance is generated proximally in the pathway where the inner wall of SC and the adjacent TM tissue interface, known as the juxtacanalicular region.^10^^,^^11^ Cells here respond homeostatically by mechanotransduction to changes in IOP by regulating resistance to aqueous humor outflow.^12^^–^^16^ However, the biochemical mechanisms involved in mechanotransduction are not well-understood.

Caveolae are specialized cellular domains that form “cup-shaped” invaginations in the membrane.^17^^,^^18^ Implicated in a variety of physiologic processes, such as membrane trafficking,^19^^–^^21^ lipid and cholesterol regulation,^22^^,^^23^ cellular signaling pathways,^24^^–^^26^ mechanotransduction,^27^^–^^29^ and mechanoprotection,^30^^,^^31^ caveolae are abundantly expressed in cells of the outflow pathway such as the SC and the TM.^11^^,^^32^ Caveolin-1 (Cav1) and caveolin-2 (Cav2) are protein scaffolds integral to the formation of caveolae, and ablation of Cav1 results in loss of caveolae.^33^ In genetic association studies, polymorphisms at the Cav1/2 gene loci have been reproducibly implicated in POAG and ocular hypertension.^34^^–^^42^ Using global Cav1 null mice, a functional link between Cav1 expression, IOP, and CO dysfunction was found.^43^^–^^45^ However, the specific location or cell type responsible for Cav1-dependent regulation of CO is unclear. A prime candidate is CO endothelia because Cav1 and caveolae mediate flow-dependent mechanotransduction in blood vessels via endothelial nitric oxide synthase (eNOS), an enzyme involved in the biosynthesis of nitric oxide (NO).^29^ NO decreases TM and SC cell volume and facilitates cell relaxation, increasing outflow facility,^46^^–^^48^ and compounds that donate NO elevate outflow facility and decrease the IOP in rabbit,^49^^–^^51^ dog,^50^ monkey,^52^ and human eyes.^53^^–^^55^ Moreover, SC endothelia release NO in response to shear stress, which leads to increased outflow through the CO pathway.^16^

Therefore, we hypothesized that caveolae in SC and the vessels distal to the SC regulate IOP and pressure-dependent aqueous humor outflow via eNOS signaling. To test this hypothesis, we created a mouse model with a targeted deletion of Cav1 in endothelial cells (herein referred to as Cav1^ΔEC^). We observed that Cav1 deficiency resulted in an elevated IOP, enlargement of vessels distal to the SC, and eNOS hyperactivity. Morphologic analyses revealed that Cav-1 deficiency did not result in significant structural defects to proximal structures of the pathway or changes in outflow facility, suggesting that the effects observed were not due to developmental deformities. Work here provides evidence that SC and distal vessels are impacted by absence of Cav1 with hyperactive eNOS signaling, and that caveolae components in the TM are important for maintaining CO function.

## Methods

### Animals

Global Cav1 knockout (KO) mice on the C57Bl/6J background (stock number 007083, Jackson Laboratory, Bar Harbor, ME) were maintained by heterozygous crosses generating wild type (WT) and KO littermates for experiments as previously described.^43^^,^^56^^–^^58^ Endothelial cell-specific Cav-1 null (Cav1^ΔEC^) mice^59^ were generated by crossing hemizygous Tie2-Cre^+/−^ mice (B6.Cg-Tg(Tek-cre)1Ywa/J; stock number 008863, Jackson Laboratory) with homozygous Cav1^lox/lox^ mice on C57BL6/J background.^60^^,^^61^ The floxed allele was bred to homozygosity with the Cre allele maintained in hemizygous state resulting in Cre-positive KO mice and Cre-negative, homozygous floxed littermates. Single-cell RNA sequencing of outflow pathway tissues from both humans^62^ and mice^63^ has definitively shown that Tie2 is expressed in SC and not in TM, thus, Cre activation only occurs in SC and vascular endothelium. Both male and female littermate mice were used for experiments. All studies involving animals were compliant with the ARVO Statement for the User of Animals in Ophthalmic and Vision Research and the National Institutes of Health Guide for the Care and Use of Laboratory Animals and were approved by the Institutional Animal Care and Use Committee of the University of Oklahoma Health Sciences Center.

Genotyping transgenic mice was done in the NEI-supported Genotyping Core (P30 EY021725) at the University of Oklahoma Health Sciences Center. For genotyping Cav1^ΔEC^ littermate mice, the following primers were used: *Cav1 flox* forward 5'-TTC TGT GTG CAA GCC TTT CC-3’; *Cav1 flox* reverse 5’-GTG TGC GCG TCA TAC ACT TG-3’; *Cre* forward 5’-AGG TGT AGA GAA GGC ACT TAG C-3’; *Cre* reverse 5’-CTA ATC GCC ATC TTC CAG CAG G-3’. For genotyping Cav1 global KO littermate mice, the following primers were used: *Cav1 WT* forward: 5’-GTG TAT GAC GCG CAC ACC AAG-3’; *Cav1 KO* forward 5’-CTA GTG AGA CGT GCT ACT TCC-3; *Cav1 common* reverse 5’-CTT GAG TTC TGT TAG CCC AG-3’.

### IOP Measurements

For IOP measurements, 8- to 12-week-old mice were anesthetized with isoflurane (3% v/v for induction; 1.0%–1.5% v/v for maintenance using a mouse nose cone). IOP was measured in both eyes within one minute of isoflurane anesthesia induction by a commercially available rodent rebound tonometer (TonoLab; ICare, Espoo, Finland) as previously described and per manufacturer's instructions.^16^^,^^43^ Pressures were consistently measured between 9 AM and noon, central time (2–5 hours after lights on in the vivarium).

### Outflow Facility Measurements

Mice were euthanized by CO_2_ inhalation followed by cervical dislocation at the end of the day (between 6 and 7 PM, Central Time) and eyes were immediately placed in DMEM (Life Technologies, Grand Island, NY) containing low glucose. Eyes were shipped (FedEx, Priority Overnight) from Oklahoma City, Oklahoma, to Durham, North Carolina, for early morning arrival (between 7:30 and 8:30 AM, Eastern Time). Immediately upon arrival, each eye was mounted on a stabilization platform in a perfusion chamber using a small amount of cyanoacrylate glue (Loctite, Westlake, OH). The perfusion chamber was filled with prewarmed D-glucose in phosphate-buffered saline (DBG, 5.5 mM) and temperature regulated at 35°C. A glass microneedle, back filled with either 10 µM L-nitro-arginine methyl ester (L-NAME) (Cayman Chemical, Ann Arbor, MI) in DBG or DBG, was connected to the system and the microneedle was inserted into anterior chamber using a micromanipulator, visualized under stereomicroscope. Outflow facility was measured with the iPerfusion system, which is especially designed to measure low outflow facilities in paired mouse eyes.^64^ First, both eyes were perfused at 12 mm Hg for 60 minutes to allow acclimatization and delivery of the drug to the outflow pathway cells, followed by nine sequential pressure steps of 4.5, 6.0, 7.5, 9.0, 10.5, 12.0, 15.0, 18.0, and 21.0 mm Hg. Data analysis was carried out as described previously.^64^ Briefly, a nonlinear flow–pressure model was used to account for the pressure dependence of outflow facility in mice. The order (left vs, right and drug vs. vehicle) in which eye perfusions were performed was randomized and identity of perfusion media (DBG vs. L-NAME) was masked.

### Immunohistochemistry and Histology

Cav1^ΔEC^ and WT mice were euthanized, eyes were enucleated, fixed with 4% paraformaldehyde, hemisected, and anterior segment wholemounts were prepared as previously described.^9^^,^^43^ Anterior segments were permeabilized with 1% Triton X-100 in PBS, blocked with 10% normal horse serum in 0.1% Triton X-100 in PBS, and incubated with the following primary antibodies at 4 °C overnight: Armenian Hamster monoclonal anti-CD31 (clone 2H8; 1:100; Developmental Studies Hybridoma Bank, University of Iowa^65^), Cy3-conjugated mouse monoclonal anti–α-smooth muscle actin (α-SMA; clone 1A4; 1:200; Sigma-Aldrich, St. Louis, MO); rabbit monoclonal anti-Cav1 (D46G3; 1:400; Cell Signaling Technology, Danvers, MA). After washing with 0.1% Triton X-100 in PBS (3 × 15 minutes), wholemounts were incubated with the appropriate fluorophore-conjugated secondary antibodies (1:500; Life Technologies, Carlsbad, CA; and/or Jackson ImmunoResearch Laboratories, West Grove, PA) at 4°C overnight. After another round of washing (3 × 15 minutes) with 0.1% Triton X-100 in PBS, 4 to 5 radial incisions were made in the anterior segments for flat-mounting in glycerol:PBS (1:1, v/v). Imaging was performed using the FV1200 confocal laser scanning microscope (Olympus, Tokyo, Japan), and images were processed with Adobe Photoshop CC 2019 (Adobe Systems, San Jose, CA). Vessel identity was established based on several criteria. Limbal venous vessels (distal to SC and to the limbal capillary plexus) were identified by connection to the SC via collector channel, by smooth muscle actin coverage, and by appearance of CD31 immunoreactivity. Limbal arterioles were distinguishable from limbal venules by the appearance of smooth muscle coverage, endothelial morphology (polarity of CD31 immunoreactivity showing alignment of endothelial cells parallel to longitudinal vessel profile), and a lack of collector channels. Limbal capillary loops were easily identifiable as they have small-caliber lumens, connect arterioles to venules, and were devoid of α-SMA immunoreactivity. Vessel diameters were measured as described^66^ using ImageJ by applying a grid and measuring vessel widths at each point where a grid line crossed the vessel. Individual vessel measurements were averaged to give an average vessel width per whole mount image.

For the Cav1^ΔEC^ and WT semithin sections, whole eyes were fixed in 4% paraformaldehyde in PBS overnight at 4°C, then transferred to PBS and shipped from the Elliott laboratory (Dean McGee Eye Institute Oklahoma City, OK) to the Stamer laboratory (Duke University Eye Center, Durham, NC) on ice. Anterior segments were divided into four quadrants, cut into 0.5 µm semithin sections, and stained in 1% methylene blue. Images were obtained using light microscopy (Axioplan2; Carl Zeiss MicroImaging, Jena, Germany).

To assess Cav1 deletion from SC but not TM, paraffin sections of outflow tissues were prepared for immunohistochemistry by fixing whole eyes in Prefer fixative (Anatech, Ltd., Battle Creek, MI) as previously described.^43^ Deparaffinized sections were blocked and stained with antibodies against Cav1 (rabbit mAB#3267, Cell Signaling Technology), CD31 (rat anti-mouse CD31, clone MEC 13.3, BD Pharmingen, Franklin Lakes, NJ), and α-SMA (Cy3-conjugated mouse monoclonal, clone 1A4, Sigma-Aldrich). Cav1 and CD31 immunoreactivity were detected by secondary antibodies conjugated with Alexafluor-488 and Alexafluor-647, respectively. Image stacks were captured on the FV1200 confocal microscopy system. Single slice images were used to generate density profiles for each color channel along lines drawn across the CO tract in Image J (see Supplementary Fig. S2A–D). Three separate profiles from nonoverlapping lines were generated for each image from *n* = 2 WT and *n* = 3 Cav1^ΔEC^ mice. The intensity of the Cav1 and CD31 signals were determined at each pixel along each line such that total Cav1 intensity in pixels that also contained CD31 signal (“Endothelial Cav1”, see Supplementary Fig. S2) and in pixels that did not contain CD31 signal (“Non-Endothelial Cav1”) could be evaluated.

### Western Blots

Anterior segments from Cav1^ΔEC^ and WT mice were isolated and iridocorneal angle tissue prepared by dissecting the bulk of the iris and ciliary body. The remaining iridocorneal angle tissue (containing TM, SC, and iris root) was dissected from anterior segments under a stereomicroscope.^67^^–^^69^ Tissues were lysed in Tris-buffered saline (10 mmol/L Tris, pH 7.4, 150 mmol/L NaCl, 1 mmol/L EDTA) containing 60 mM octylglucoside and supplemented with protease inhibitor cocktail (Calbiochem; EMD Millipore, Burlington, MA). Protein content was determined with a Pierce BCA assay (Thermo Fisher Scientific, Rockford, IL), using bovine serum albumin as a standard. Equal amounts of protein were resolved on 4% to 20% or 4% to 12% Tris-glycine gradient gels (Life Technologies). Proteins were transferred to nitrocellulose membranes and then probed with antibodies, using standard methods. Primary antibodies and dilutions were as follows: rabbit anti-phospho-eNOS (Ser1177; 1:1000; Cell Signaling Technology); rabbit monoclonal anti-eNOS (D9A5L; 1:1000; Cell Signaling Technology); rabbit anti-nitrotyrosine (1:500; EMD Millipore); and mouse monoclonal anti-glyceraldehyde-3-phosphate dehydrogenase (1:300; EMD Millipore). Immunoreactivity was detected using horseradish peroxidase–conjugated secondary antibodies (1:5000; GE Healthcare, Little Chalfont, UK) and imaged with a Kodak In Vivo F-Pro system (Carestream, Rochester, NY).

To assess eNOS phosphorylation in response to transient IOP elevation, mice were anesthetized with 100 mg/kg ketamine/10 mg/kg xylazine and the anterior chamber of one eye was cannulated to a sterile saline-filled reservoir. The infusion set up is similar to that previously described for assessment of pressure-induced tissue injury,^43^ except that propidium iodide was not infused and IOP was elevated only to 30 mm Hg for 30 minutes in the current experiments. Immediately after IOP elevation, mice were euthanized, enucleated, and iridocorneal angle tissue was dissected.

### Statistical Analysis

Graphing and statistical analyses were performed using GraphPad Prism v.8 (GraphPad Software, La Jolla, CA). A *P* value of 0.05 or less was considered significant and data are presented as mean ± SEM, unless otherwise stated.

## Results

### Tie2/Cre Effectively Ablates Cav1 in Vascular Endothelia

**Figure 1.**
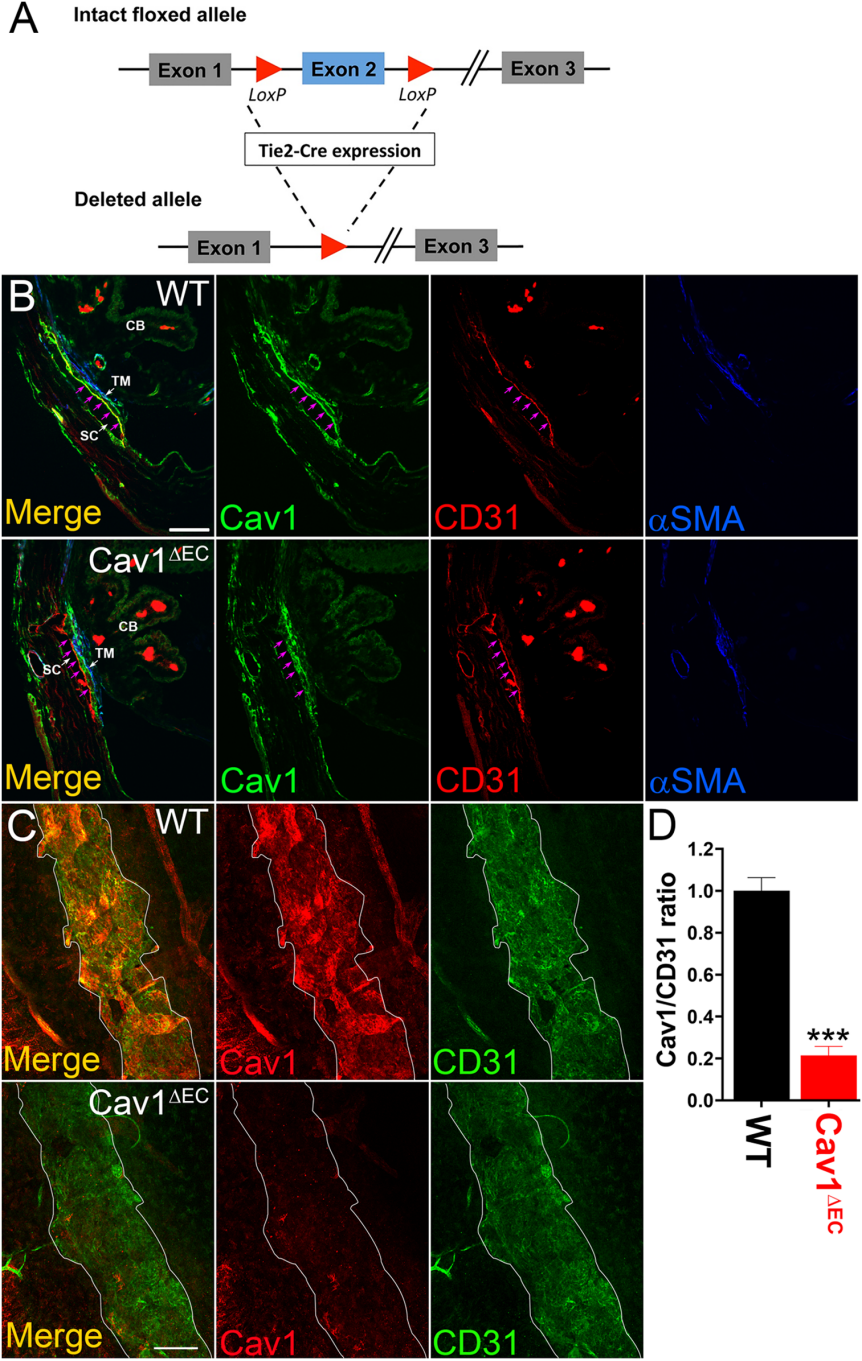
Tie2-Cre effectively ablates Cav1 from the SC, but not TM. (**A**) Schematic representation of Tie-2-Cre expression on an intact floxed allele to produce a deleted allele*.* (**B**) Transverse iridocorneal section depicting distal vessels, SC, and TM. CD31 (*red*) labels the SC and distal vessel endothelium; αSMA (*blue*) labels the TM. Cav1 (*green*) is expressed in the SC and in the TM of the WT mouse CO pathway (*top*)*.* In Cav1^ΔEC^, Cav-1 is only expressed in the TM, and not the SC (*bottom*). See *magenta arrows* outlining inner wall of SC. Scale bar, 50µm. (**C**) En face images of a confocal slice through the SC region of anterior segment whole mounts. *White border* outlines SC. Cav1 immunoreactivity (*red*) in the SC is strong in the WT mouse, but absent from the SC of the Cav1^ΔEC^. CD31 immunoreactivity (*green*) represents SC. Scale bar, 100µm. (**D**) Densitometry analysis depicting Cav1/CD31 ratio from iridocorneal images in panel C. There was a significant reduction in Cav1/CD31 labelling in Cav1^ΔEC^ mice compared with WT control mice (^****^P < 0.0001, unpaired *t* test analysis, *n* = 4 for each group).

Using Tie2 promoter-driven Cre-mediated recombination, exon 2 of Cav1 was specifically recombined resulting in the ablation of Cav1 from vascular endothelia including SC (Fig. 1A). To confirm deletion of Cav1, we examined isolated tissues from brain, heart, lung, liver, kidney, and fat by immunoblotting with anti-Cav1-specific IgG (Supplementary Fig. S1). In highly vascularized tissues such as brain, heart, lung, and liver, Cav1 depletion was robust. Endothelial Cav1 ablation was not obvious by Western blotting of nonvascular tissues in which Cav1 is highly expressed such as kidney and fat, suggesting selective ablation of Cav1 from vasculature. As a control, we examined tissues for caveolin-3, which is predominantly expressed in muscle tissue and 65% identical to Cav1.^70^ We found that caveolin-3 is expressed at similar levels in cardiac tissue of both Cav1^ΔEC^ and WT mice (Supplementary Fig. S1).

**Figure 2.**
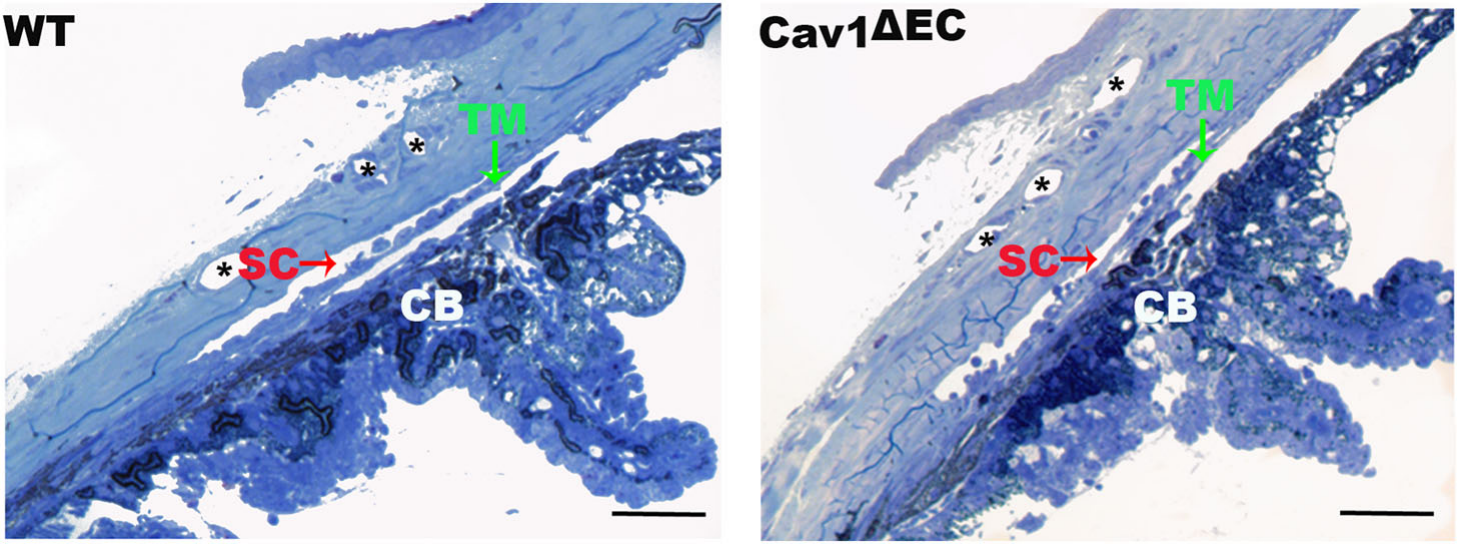
Structural analysis of CO pathway in Cav1^ΔEC^ and WT**.** Light microscopy of iridocorneal angle structures in semi-thin sections of WT (*left*) and Cav1^ΔEC^ (*right*) mice stained with methylene blue. The angle is open in both genotypes. There were no visible abnormalities on gross morphology in the CO pathway structures. CB, ciliary body. *Distal vessels. Scale bars = 50 µm.

Our Cav1^ΔEC^ mouse model provides a unique opportunity to test the specific contribution of endothelial Cav1 in the regulation of CO because our previous work uncovered a functional link between global Cav1 KO, and CO dysfunction involving NO.^43^ Thus, we next determined whether Cav1 was effectively ablated from the vascular endothelia of the CO pathway. The two main cell types of the proximal portion of the CO pathway, the TM cells and SC endothelium abundantly express Cav1 and contain numerous caveolae.^32^^,^^43^ We labeled iridocorneal angle tissues from Cav1^ΔEC^ and WT mice with antibodies against Cav1, CD31, and, α-SMA. We used CD31 as endothelial and α-SMA as smooth muscle markers, respectively. In Cav1^ΔEC^ mice, we did not detect significant Cav1 expression in SC or endothelial cells of distal CO vessels and ciliary body. In contrast, we observed Cav1 expression in nonendothelial cells such as in the TM and ciliary muscle (Fig. 1). Outflow tissues were analyzed in sagittal sections (Fig. 1B) and anterior segment wholemount preparations (Fig. 1C). In control mice, we found that Cav1 was localized to the SC and TM, whereas in Cav1^ΔEC^ mice, Cav1 was visible in the TM and absent in the SC (Fig. 1B). In confocal images of SC, en face (Fig. 1C), both Cav1 (red) and the endothelial marker CD31 (green) colocalize in WT but Cav1 immunoreactivity was dramatically reduced in Cav1^ΔEC^ SC (Fig. 1C). Densitometric image analysis of the ratio of Cav1/CD31 immunoreactivity in the SC region showed a significant 79% decrease in the Cav1/CD31 ratio in the SC of Cav1^ΔEC^ compared with WT (Fig. 1D). To further evaluate the efficiency and specificity of Cav1 deletion in the SC, we analyzed individual confocal slices from sagittal paraffin sections immunostained for Cav1, CD31, and α-SMA. To do so quantitatively, we examined the intensity of Cav1 signal in CD31-positive and CD31-negative pixels along lines spanning the SC/TM region as described in the Methods. As shown in Supplementary Figure S2A–D, the SC inner walls and outer walls and the TM can be distinguished. A subset of TM that is α-SMA positive is shown in blue. Note in the representative profile from a WT mouse shown in Supplementary Figure S2B that there are Cav1-positive peaks aligning with the inner walls and outer walls, but this inner wall- and outer wall-associated Cav1 signal is largely absent in the Cav1^ΔEC^ profile. By dividing three replicate profiles from *n* = 2 WT and *n* = 3 Cav1^ΔEC^ mice determined the total Cav1 intensity in CD31-positive (“Endothelial Cav1”; Supplementary Fig, S2E) and CD31 negative (“Non-endothelial Cav1”; Supplementary Fig. S2F). We observed a significant approximately 72% decrease in endothelial Cav1, but no change in nonendothelial Cav1 using this independent evaluation, which agrees nicely with our deletion efficiency determine analysis from whole mount staining (Fig. 1C, D). Both of these analyses likely underestimate the deletion efficiency given the abundant expression of Cav1 in the adjacent TM. Overall, these results confirm that Tie2-Cre effectively ablates Cav1 from SC and other vascular endothelial cells, but not TM or other nonendothelial tissue. Importantly, this efficient ablation of Cav1 from vascular endothelia did not result in gross morphologic changes to the SC, TM, or ciliary body (Fig. 2).

### Endothelium-Specific Cav1 Ablation Results in Ocular Hypertension

**Figure 3.**
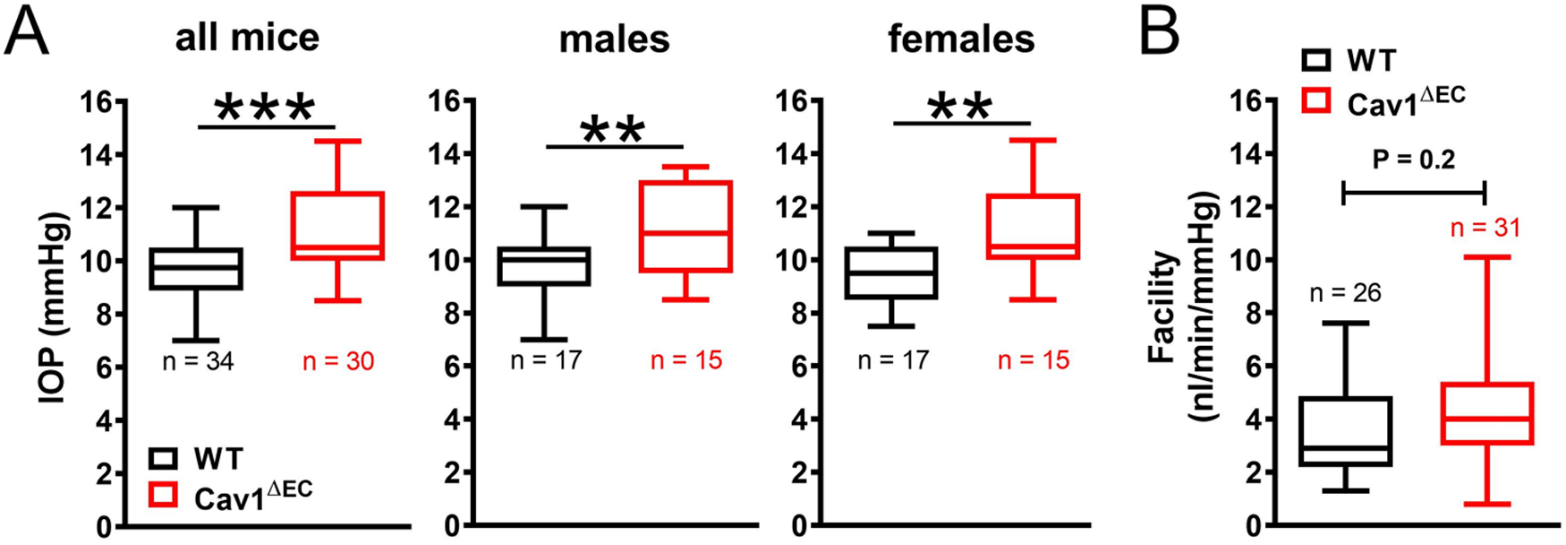
IOP and outflow facility in Cav1^ΔEC^ mice. (**A**) The IOP was significantly increased in Cav1^ΔEC^ mice compared with WT mice, as measured by rebound tonometry (^***^P = 0.0001, two-tailed unpaired *t* test analysis, *n* = 30–34 mice for each group). IOP was also significantly increased in both male and female Cav1^ΔEC^ mice compared with WT mice (^**^P = 0.0075, two-tailed unpaired *t* test analysis, *n* = 15–17 mice for each group). (**B**) Outflow facility not significantly different between Cav1^ΔEC^ mice and WT mice (*P* = 0.2, two-tailed unpaired *t* test analysis, *n* = 26–31 mice for each group).

Cav1 is abundant in human and murine CO tissues, including the SC and distal vessels.^43^ In mice in which Cav1 is deleted globally, the IOP is elevated and outflow facility is concomitantly reduced compared with controls.^43^^–^^45^ To determine whether the observed ocular hypertension and dysfunctional pressure-dependent CO homeostasis resulted from loss of Cav1 expression and function specifically in the SC and/or distal vascular endothelium, we measured IOP and outflow facility in Cav1^ΔEC^ mice. As with the global KOs, the IOP was significantly increased in Cav1^ΔEC^ mice (11.1 ± 0.3 mm Hg) compared with WT mice (9.6 ± 0.2 mm Hg) (Fig. 3A, *P* = 0.0001, *n* = 30–34 mice for each group). Because some Cav1 single nucleotide polymorphisms are more strongly associated with POAG in females,^38^ we investigated whether there was sexual dimorphism in IOP readings between male and female Cav1^ΔEC^ mice. IOP was similarly increased in Cav1^ΔEC^ male (11.1 ± 0.4 mm Hg) and female (11.0 ± 0.5 mm Hg) mice compared with WT control male (9.7 ± 0.3 mm Hg) and female (9.6 ± 0.3 mm Hg) mice (Fig. 3A, *P* = 0.0075, *n* = 15–17 mice for each group).

To determine whether elevated IOP in Cav1^ΔEC^ mice resulted from increased resistance (decreased facility) to aqueous humor drainage in the CO pathway, we measured flow while maintaining sequential pressures in perfused Cav1^ΔEC^ and WT eyes as previously described.^16^^,^^43^^,^^71^^,^^72^ Surprisingly, outflow facility was not significantly different between Cav1^ΔEC^ mice (5.3 ± 0.6 nL/min/mm Hg) and WT mice (3.8 ± 0.5 nL/min/mm Hg), although there was a trend toward increased outflow facility in the endo-KO mice (Fig. 3B, *P* = 0.2, *n* = 26–31 mice for each group). Thus, increased resistance to CO in the TM/SC cannot explain ocular hypertension resulting from ablation of endothelial Cav1. In fact, Cav1 expression by TM seems to rescue dysfunctional CO. Regardless, there is likely to be another mechanism whereby ablation of endothelial Cav1 results in ocular hypertension.

### Endothelial Cav1 Mediates NO Activity in the CO Pathway

**Figure 4.**
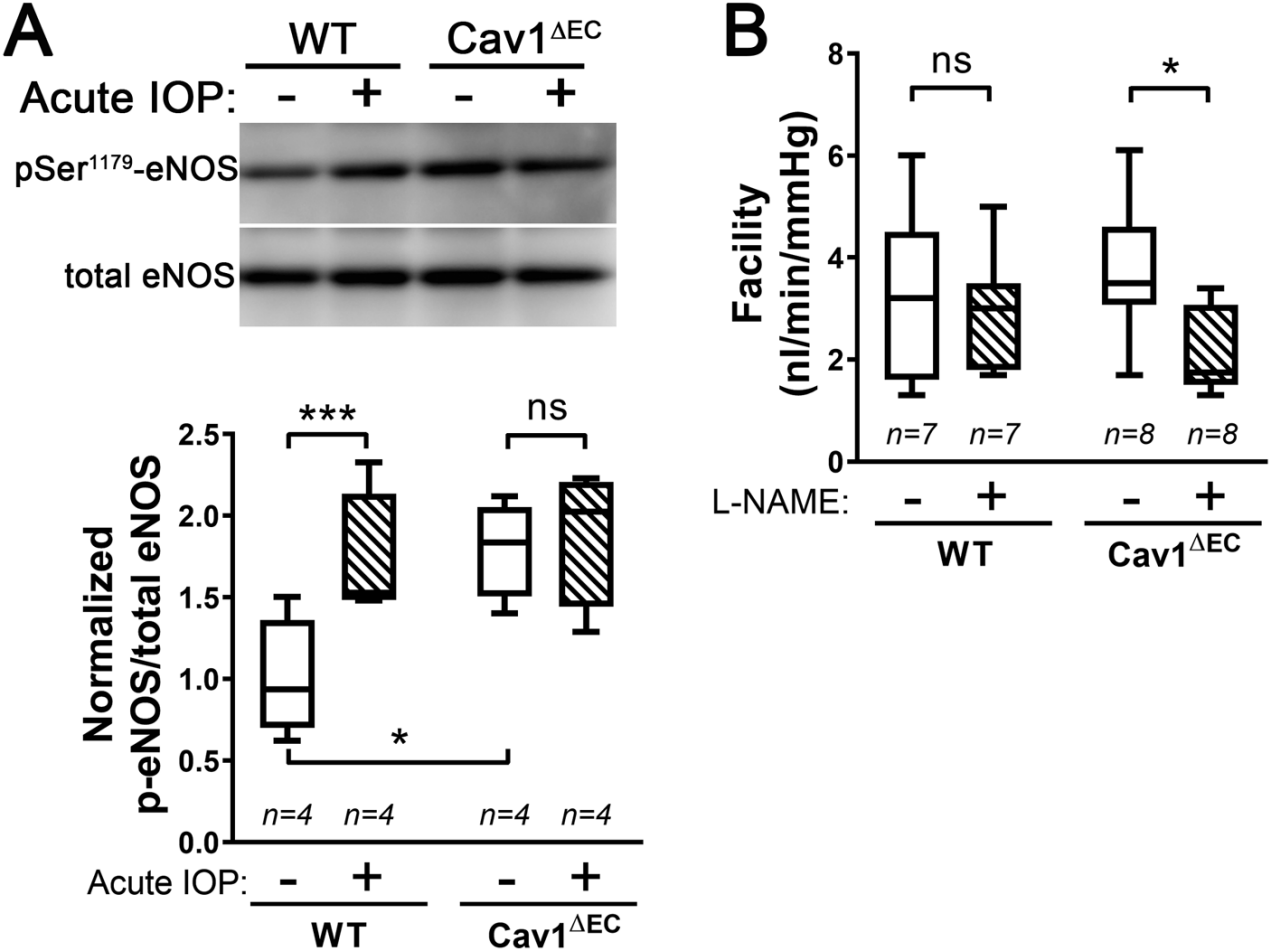
Deficiency of endothelial caveolae results in eNOS hyperactivity in the CO pathway. (**A**) Immunoblot depicting eNOS and phosphorylated eNOS (p-eNOS) expression in iridocorneal tissue from WT and Cav1^ΔEC^ mice (**A***, top*). In WT, p-eNOS expression was significantly higher in protein lysates from mice with an acute 30 mm Hg IOP elevation (*n* = 4) compared with mice with no IOP elevation (*n* = 4, *P* < 0.001, repeated measure two-way ANOVA, Sidak's post hoc test). As well, p-eNOS expression was significantly increased in Cav1^ΔEC^ with no IOP elevation compared with WT with no IOP elevation (*P* = 0.02, repeated measure two-way ANOVA, Sidak's post hoc test) (**A**, *bottom*). In Cav1^ΔEC^ mice, no change in p-eNOS expression was observed between acute IOP elevation (*n* = 4) and no IOP elevation (*n* = 4) (repeated measure two-way ANOVA, Sidak's post hoc test). (B) Outflow facility was measured in perfused, enucleated eyes subjected to sequential pressure steps. L-NAME treatment had no effect on conventional, pressure-dependent outflow in WT eyes (*n* = 7) compared with no drug treatment (*n* = 7). Contrastingly, outflow facility was significantly reduced in Cav1^ΔEC^ eyes treated with L-NAME (*n* = 8) compared with no drug treatment (*n* = 8) (^*^P < 0.05, *n* = 7–8, ratio paired *t* test).

Global Cav1 KO results in elevated IOP and decreased outflow facility in mice, despite increased eNOS activity.^43^ The specific tissue attributable to the effects of Cav1 knockdown and subsequent eNOS hyperactivity in the CO pathway is still unknown. We hypothesized that endothelial Cav1 is involved in the eNOS-dependent transduction of mechanical signals in the CO pathway, such as changes in IOP. To investigate this hypothesis, we tested whether endothelial Cav1 deletion influenced phosphorylation at the activating serine-1177 position of eNOS. We assessed eNOS phosphorylation before and after acute elevation of IOP in iridocorneal angle tissues isolated immediately after a 30 minutes experimental elevation of IOP in vivo (Fig. 4A). In WT, phosphorylated eNOS was higher in protein samples from acute IOP elevation (1.7 ± 0.2) compared with normotensive eyes (1.0 ± 0.2, *P* = 0.04, *n* = 4), suggesting acute IOP activated eNOS in the CO pathway, as previously described.^16^ In contrast, no change in phosphorylated eNOS was observed when the IOP was acutely elevated in Cav1^ΔEC^ mice (1.9 ± 0.2 vs. 1.8 ± 0.1, *P* = 0.9, *n* = 4), suggesting that the eNOS-mediated transduction of mechanical signals requires Cav1 in the CO pathway. Not surprisingly, basal eNOS activation was higher Cav1^ΔEC^ than WT mice (*P* = 0.02, *n* = 4), supporting previous reports in other tissues of eNOS hyperactivity in the absence of caveolae.^33^

We then tested whether outflow facility in Cav1^ΔEC^ mouse eyes was more sensitive to eNOS inhibition as shown in global Cav1 KO mouse eyes^43^ (Fig. 4B). We measured changes in flow in the face of sequential pressure steps in enucleated WT and Cav1^ΔEC^ eyes following treatment with and without eNOS inhibitor, L-NAME (10 µM) (Fig. 4B). In WT mice, L-NAME treatment had no significant effect on outflow facility (3.0 ± 0.4 nL/min/mm Hg) as compared with untreated (3.3 ± 0.6 nL/min/mm Hg, *P* > 0.8, *n* = 7). In Cav1^ΔEC^ mice, L-NAME treatment significantly reduced outflow facility (2.1 ± 0.3 nL/min/mm Hg) as compared with untreated (3.7 ± 0.5 nL/min/mm Hg, *P* < 0.05, *n* = 8). These data were similar to the effects observed in global Cav1-KO mice,^43^ and suggest that eNOS hyperactivity in the CO pathway is at least partly attributable to caveolae deficiency in the endothelial cells of the SC and/or the distal vessels.

### Enlarged Distal CO Vessels in Cav1^ΔEC^ and Global Cav1 KO in Mice

**Figure 5.**
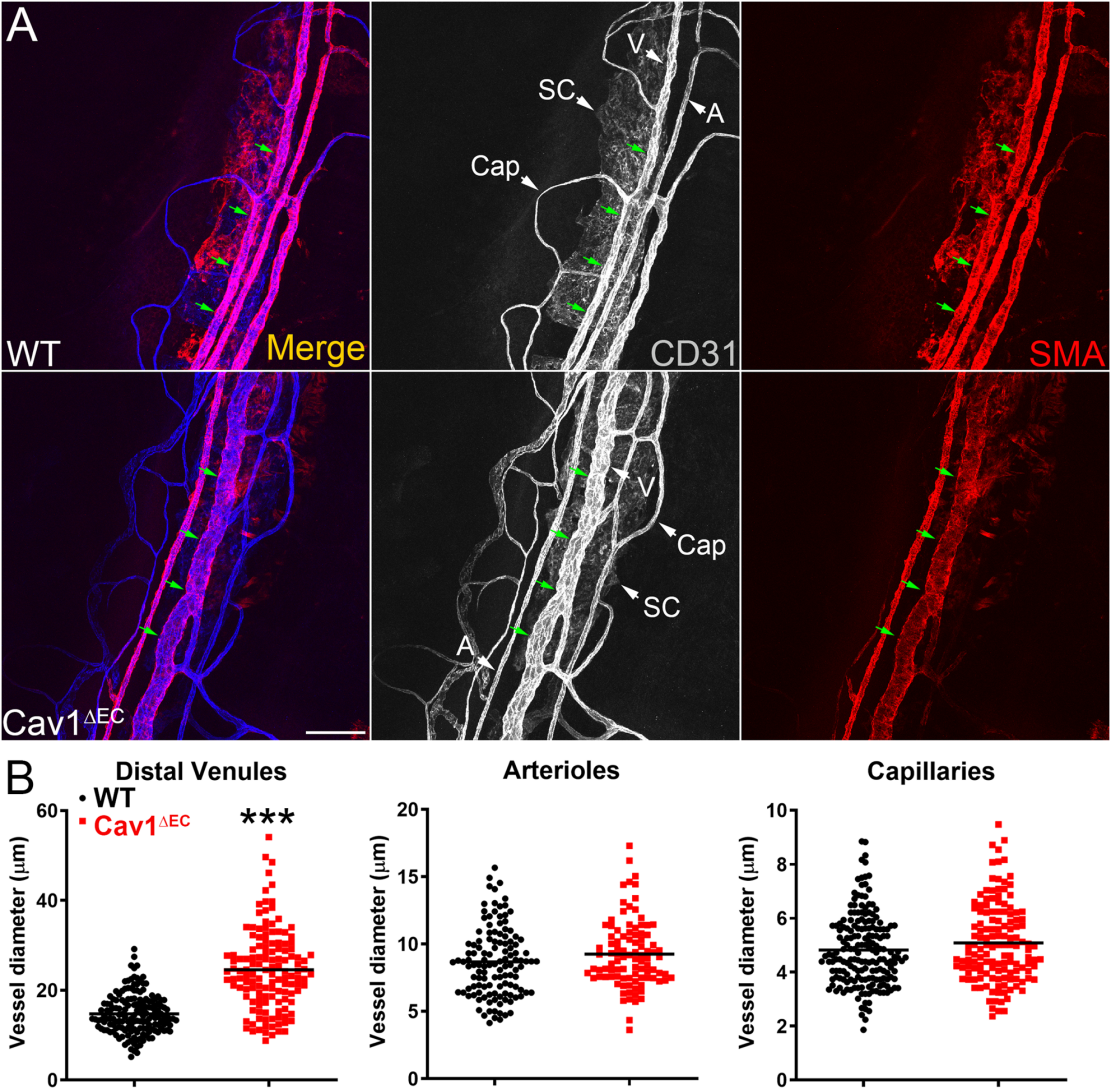
Distal vessel enlargement in Cav1^ΔEC^ mice. (**A**) Confocal microscopic projections of limbal vasculature from WT (*top row*) and Cav1^ΔEC^ (*bottom row*) mice. Immunofluorescent labelling of CD31 (*white*) stains for all vasculature and α-SMA (*red*) stains for all distal vessels except capillaries; merged stains shown in left-most column. Additionally, distal venule enlargement is visible on Cav1^ΔEC^ tissue compared with WT (*green arrows*). Scale bar = 100 µm. A, arteriole; Cap, capillary; V, venule. (**B**) Quantitative analyses of distal vessel diameters in venules, arterioles, and capillaries. Vessel diameter of distal arterioles and capillaries was not significantly different between WT (*n* = 8 mice) and Cav1^ΔEC^ (*n* = 7 mice). Vessel diameter of distal venules was significantly increased in Cav1^ΔEC^ (*n* = 7 mice) compared with WT (*n* = 8 mice) (^***^P = 0.0002, unpaired two-tailed *t* test). Each point represents a single measurement along the vessel. For statistical analysis, each *n* value is calculated as the mean of all measurements along a vessel region for each mouse.

Results thus far indicate that eNOS signaling is hyperactive in endothelial Cav1 KO mice, and that endothelial Cav1 and eNOS play a coordinated role in the mechanotransduction and regulation of pressure changes within the CO pathway. Thus, we hypothesized that hyperactive eNOS signaling owing to Cav1 deficiency in the vessels distal to SC would result in an increased vessel diameter, owing to persistent NO-mediated vasodilation.^73^ We examined limbal vasculature stained for α-SMA, and CD31 (Supplementary Fig. 3A and Fig. 5A) in anterior segment wholemounts by confocal microscopy. We then quantified vessel diameters in multiple locations along venules, arterioles, and capillaries, to estimate the mean vessel diameter for each vessel type. In Cav1^ΔEC^ mice, α-SMA and CD31 staining clearly showed distal venule enlargement as compared with WT mice (Fig. 5A). In Cav1^ΔEC^ mice, the distal venule mean diameter (25.8 ± 2.0 µm) was significantly greater than that of WT mice (14.7 ± 0.1 µm, *P* = 0.0002, *n* = 7–8). There were no significant changes in arteriolar diameter (Cav1^ΔEC^, 8.9 ± 0.5 µm; WT, 8.5 ± 0.5 µm, *P* = 0.65, *n* = 7–8) or capillary diameter (Cav1^ΔEC^, 4.9 ± 0.3 µm; WT, 4.8 ± 0.1 µm, *P* = 0.89, *n* = 7–8) between genotypes. We went back and examined our global Cav1 KO mice and found similar distal venous enlargement compared with WT littermates (Supplementary Fig. 3A). In global Cav1 KO mice, the distal venule mean diameter (26 ± 2.0 µm) was significantly greater than that of WT (11.4 ± 1.5 µm, *P* = 0.0004, *n* = 5–6). Unlike Cav1^ΔEC^, global Cav1 KO mice also exhibited a modest but significant enlargement of capillary diameter as well (5.3 ± 0.2 µm) as compared with WT (4.5 ± 0.2 µm, *P* = 0.04, *n* = 5–6) (Supplementary Fig. 3B). Therefore, endothelial KO of Cav1 results in distal venule but not arteriole or capillary enlargement in the CO pathway.

### Increased Tyrosine Nitration in Anterior Segments of Cav1^ΔEC^ Mice

**Figure 6.**
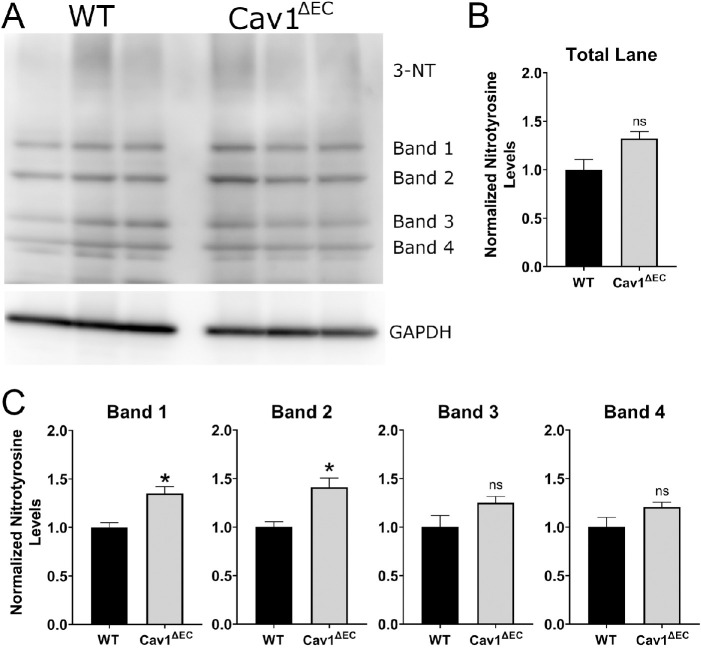
Densitometric studies of nitrotyrosine levels in Cav1^ΔEC^ mice. (**A**) Immunoblots staining for nitrotyrosine (3-NT) in protein lysates extracted from iridocorneal angles of WT and Cav1^ΔEC^ mice. Each column represents protein extracted from a single mouse (*n* = 3 for each group). There are four main bands evident. All signal intensities were normalized to glyceraldehyde-3-phosphate dehydrogenase. (**B**) Densitometric analysis depicting data for each separate band within each sample. There was a significant increase in tyrosine nitration of the Cav1^ΔEC^ compared with the WT samples for band 1 (*P* = 0.015) and band 2 (*P* = 0.019). There was no significant difference between Cav1^ΔEC^ and WT for bands 3 and 4. (**C**) Densitometric analysis depicting combined data for all four bands within each lane, for each group. When calculating total nitrotyrosine levels, there was no significant difference between WT and Cav1^ΔEC^. All data were analyzed using unpaired *t* test, *n* = 3 for all groups. All densitometry data was normalized to WT control.

We observed elevated IOP in Cav1^ΔEC^ mice despite increased eNOS activity. This finding seems to be contradictory to previous work showing that NO enhances CO, subsequently lowering IOP.^16^^,^^51^^,^^52^^,^^74^ Therefore, we sought an explanation to reconcile these two findings. We hypothesized that chronically elevated NO levels secondary to Cav1 ablation were causing toxicity owing to excessive nitration in the outflow pathway, ultimately contributing to elevated IOP. Because detrimental effects of protein nitration are detectable via measurement of nitrotyrosine levels,^75^^–^^79^ we performed immunoblotting for nitrotyrosine in samples from Cav1^ΔEC^ and WT mice, and standardized to glyceraldehyde-3-phosphate dehydrogenase (Fig. 6A). Four major bands were evident when immunoblotting for nitrotyrosine. When combining the densitometry analysis for all four bands, there was no significant difference between the levels of nitrotyrosine in WT (0.30 ± 0.03) compared with Cav1^ΔEC^ (0.39 ± 0.02) (Fig. 6B, *P* = 0.07, *n* = 3). When densitometry was conducted for each of the four bands (Fig. 6C), nitrotyrosine levels for bands 3 and 4 were elevated Cav1^ΔEC^ compared with WT, although not significantly (*P* = 0.14, *n* = 4). However, nitrotyrosine levels Cav1^ΔEC^ were significantly elevated for band 1 (35% increase, *P* = 0.02, *n* = 3) and band 2 (41% increase, *P* = 0.02, *n* = 3), as compared with WT. Thus, there is evidence for increased nitration of some proteins in Cav1^ΔEC^ mice, as compared with WT. Interestingly, we did not observe significant differences in nitrotyrosine levels in global Cav1 KO, although a trend towards elevation was noticed (Supplementary Fig. 4).

### Decreased Outflow Facility and Distal Venule Diameter Following Endothelin-1 (ET-1) Treatment in WT and Cav1^ΔEC^ Mice

**Figure 7.**
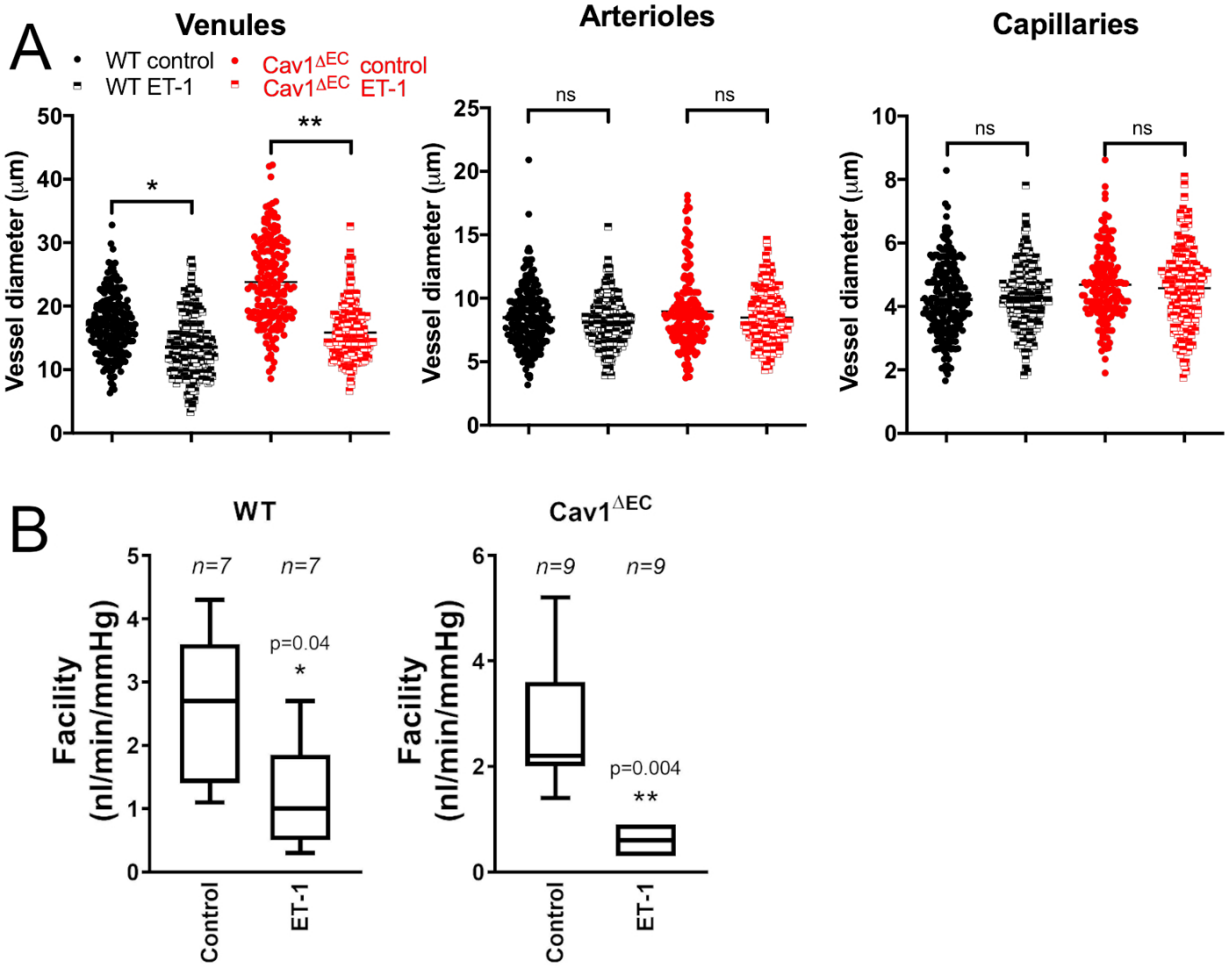
Decreased distal vessel size and reduced outflow facility after ET-1 treatment in WT and Cav1^ΔEC^ mice. (**A**) Quantitative analyses of distal vessel diameters in venules, arterioles, and capillaries after treatment with ET-1. No significant change of vessel diameter was observable in arterioles or capillaries of the WT (*n* = 6) or Cav1^ΔEC^ (*n* = 5) mice after ET-1 treatment. Vessel diameter of distal venules was significantly decreased in both WT (*n* = 6, ^*^P = 0.02, ratio paired *t* test) and Cav1^ΔEC^ (*n* = 5, ^**^P = 0.002, ratio paired *t* test) mice after ET-1 treatment. Each point represents a single measurement along the vessel. For statistical analysis, each *n* value is calculated as the mean of all measurements along a vessel region for each mouse. (**B**) Outflow facility was significantly decreased after ET-1 (100 nM) treatment in both WT (*n* = 7, ^*^P = 0.04, paired ratio *t* test) and Cav1^ΔEC^ (*n* = 9, ^**^P = 0.004, paired ratio *t* test) mice. ns, not significant.

To confirm the observed differences between WT and Cav1^ΔEC^ mice were not due to permanent structural changes in the distal vessels, we perfused eyes with the vasoconstrictor peptide ET-1 and measured distal vessel diameters (Fig. 7A) and outflow facility (Fig. 7B). In WT mice, the distal venule mean diameter (17 ± 1.1 µm) was significantly decreased after perfusion with ET-1 (14 ± 0.87 µm, *P* = 0.02, *n* = 6). In Cav1^ΔEC^ mice, ET-1 more potently reduced the mean distal venule diameter (24 ± 0.67 µm) after perfusion with ET-1 (16 ± 0.7 µm, *P* = 0.002, *n* = 5). There were no significant changes of arteriolar or capillary diameters following ET-1 treatment in WT or Cav1^ΔEC^ mice. Outflow facility was significantly reduced after ET-1 treatment in both WT and Cav1^ΔEC^ mice. In WT mice, ET-1 treatment significantly decreased outflow facility (1.2 ± 0.28 nL/min/mm Hg) as compared with untreated mice (2.5 ± 0.4 nL/min/mm Hg, *P* = 0.04, *n* = 9). As observed for distal venule diameter, the effect of ET-1 on outflow facility in Cav1^ΔEC^ mice was also more potent (Fig. 7B). ET-1 treatment significantly reduced outflow facility (0.6 ± 0.1 nL/min/mm Hg) as compared with untreated mice (2.7 ± 0.48 nL/min/mm Hg, *P* = 0.004, *n* = 7). These data suggest that observed differences of distal vessel diameters between WT and Cav1^ΔEC^ mice are due to functional changes from excessive NO production, and are not due to permanent structural rearrangement.

## Discussion

Caveolae are crucial in the regulation of outflow resistance and mechanoresponsiveness of outflow tissues.^43^ Thus, it is likely that reduced Cav1 expression in CO tissues contributes to increased POAG risk in patients with risk-associated polymorphisms.^36^^,^^37^ Because caveolae are abundantly present in both major cell types of the CO pathway, we generated an endothelial-specific Cav1 KO (Cav1^ΔEC^) mouse model to evaluate the role of caveolae in SC and distal CO vessels. Here, we observed that Cav1 and caveolae, which are present in the SC and distal vessels, are important regulators of IOP, partly via mediation of NO signaling. Interestingly, we found that IOP is elevated in Cav1^ΔEC^ mice, compared with WT mice, without a concomitant decrease in outflow facility, indicating that the TM has compensated to normalize CO. We also observed that endothelial Cav1 deficiency resulted in eNOS hyperactivity, demonstrable in Cav1^ΔEC^ mice by an increased level of eNOS phosphorylation within the anterior segment, hypersensitivity of endoCav1 KO eyes to the NOS inhibitor L-NAME, and distal vessel enlargement. Additionally, we detected that chronic elevation of eNOS activity in endo Cav1-KO eyes resulted in increased nitration of tyrosine residues, which could have implications in protein dysfunction and tissue damage in the CO pathway.

Various physiologic roles of caveolin and caveolae have been implicated in the regulation of IOP and aqueous humor outflow, such as extracellular matrix remodeling, membrane mechanosensation and mechanoprotection, cytoskeletal dynamics, and regulation of eNOS signalling.^80^^,^^81^ The biosynthesis of NO requires the enzymatic activity of eNOS,^29^ and NO production in the SC seems to be an important homeostatic mechanism for regulating IOP.^16^ Elevated IOP leads to narrowing of the SC lumen and subsequent elevated shear stress imposed on SC endothelia, which triggers NO release.^82^ Elevated NO levels in the SC and the adjacent juxtacanalicular region enhances CO, thus homeostatically decreasing the IOP.^16^^,^^51^^,^^52^^,^^74^ Additionally, the vessels distal to SC account for 25% to 50% of the total outflow resistance in the CO pathway,^83^ and are responsive to NO. Hence, treatment of trabeculotomized perfused anterior segments with a NO donor significantly increases outflow facility.^54^ The efficacy of targeting the NO pathway in patients with glaucoma has been shown in multiple human studies,^84^^–^^88^ and other NO donors for use in clinical trials are in development.

Cav1 binds to and negatively regulates eNOS,^24^^,^^25^^,^^89^^,^^90^ and when intracellular levels of Ca^2+^ increase, eNOS detaches from Cav1 and is activated.^89^ Transient silencing of Cav1 in human anterior segments increases outflow facility via enhanced eNOS signaling in the SC and distal vessels.^91^ In contrast, our work provides evidence that chronic loss of endothelial Cav1 resulted in elevated IOP without a change in outflow facility, despite considerable eNOS hyperactivity and enlarged distal vessels. This result is consistent with previous findings in global Cav1 KO mouse models,^43^^–^^45^ and suggests that the effects on IOP and eNOS signaling observed are at least partly attributable to Cav1 function in the SC and distal vessels^43^ (Supplementary Fig. 3). Moreover, these findings demonstrate dramatic differences in the physiologic side effects of chronic and transient Cav1 knockdown. We propose that transient loss of Cav1 results in rapid eNOS hyperactivation and subsequent increase in outflow facility, whereas chronic loss of Cav1 and caveolae cause CO tissue dysfunction and increased IOP that is partially compensated for by eNOS hyperactivation.

Elevated levels of eNOS activity were demonstrable by an increased level of eNOS phosphorylation in the anterior segment of Cav1^ΔEC^ compared with WT tissue, and hypersensitivity of Cav1^ΔEC^ eyes to the NOS inhibitor L-NAME. Specifically, outflow facility in Cav1^ΔEC^ mouse eyes was significantly decreased in response to L-NAME treatment, whereas L-NAME had no effect on outflow facility of WT mouse eyes. eNOS hyperactivity in the SC and distal vessels seemed to be causing an increase in basal outflow facility to compensate for Cav1-mediated CO defects and subsequently elevated IOP. This theory is consistent with work in nonocular tissue showing hypersensitivity to L-NAME treatment in global Cav1 KO mice. Maniatis et al.^92^ (2008) showed that global Cav1 KO mice exhibited increased pulmonary hypertension and elevated pulmonary vascular resistance despite hyperactive eNOS and significantly higher plasma NO levels. Treatment with L-NAME significantly elevated pulmonary vascular resistance in global Cav1 KO mice compared with WT, suggesting greater NO-mediated pulmonary vasodilation in global Cav1 KO mice. Elevated pulmonary vascular resistance in global Cav1 KO mice was due to pulmonary precapillary structural abnormalities, and elevated plasma NO levels partially compensated for this by promoting vasodilation.^92^ Additionally, distal venules in Cav1^ΔEC^ mice were enlarged compared with WT, and we hypothesized that the distal venule enlargement was due to NO-mediated vasodilation.

The TM might also be detrimentally affected by excess NO, but because the TM still expresses Cav1 we theorize that it is still able to maintain homeostasis via Cav1-mediated responses to mechanical strain, explaining the lack of CO defects in Cav1^ΔEC^ mice. To determine whether long-term exposure to elevated NO levels in Cav1^ΔEC^ mice caused permanent structural changes in the distal vasculature that resulted in enlarged distal venules, we perfused WT and Cav1^ΔEC^ eyes with ET-1 and monitored the effects on distal vessel diameter and outflow facility. We found that ET-1 decreased the outflow facility and caused distal venule constriction in both WT and Cav1^ΔEC^ mice, suggesting that the dilation of distal venules observed in global and Cav1^ΔEC^ mice was a functional and not structural change. Interestingly, we could not reverse distal venule enlargement in Cav1^ΔEC^ mice with L-NAME (Supplementary Fig. 5), suggesting that short-term treatment with L-NAME was not sufficient to significantly reverse life-long NO-mediated vessel dilation. Endogenous factors controlling tone, such as ET-1, may be lower in Cav1^ΔEC^ mice owing to dominance of NO activity over time. As NO inhibits ET-1 expression and secretion,^93^^,^^94^ chronic eNOS hyperactivity secondary to endothelial Cav1 ablation may also downregulated ET-1. Removing NO production with L-NAME only inhibits vessel relaxation, but if endogenous ET-1 expression is downregulated, vessels would not constrict to the same degree. Moreover, the effect of L-NAME on decreasing outflow facility in Cav1^ΔEC^ mice might be a preferential effect of NO inhibition in areas we did not measure morphologically such as the SC or on CCs. Thus, future work will investigate how short-term conditional Cav1^ΔEC^ affects IOP, outflow facility, and distal vessel size.

An alternative explanation for endothelial Cav1-mediated CO defects is nitrative stress. Chronic elevation of NO levels leads to excessive reactivity with superoxide, which produces the oxidant and nitrating agent, peroxynitrite. Tissue damage and cell death are caused by peroxynitrite-mediated formation of nitrotyrosine,^75^^–^^79^ disrupting phosphorylation and targeting certain proteins for degradation.^77^ A limitation of our study is that we did not investigate tissue damage using electron microscopy; however, we did exhaustively conduct electron microscopy imaging of global Cav1 KO mice for our previous work and we found no evidence of tissue damage under normotensive conditions.^43^ It is unlikely that this result would be any different for Cav1^ΔEC^ mice. Furthermore, the responsive of the outflow tissue and distal vasculature of the Cav1^ΔEC^ mice to ET-1 was similar to WT mice, suggesting that tissue damage is not a major issue. In this study, we did observe that nitrotyrosine levels were significantly elevated in select proteins from Cav1^ΔEC^ mice. This finding is consistent with previous work in global Cav1 KO mice, which theorized that excessive levels of NO secondary to Cav1 KO may result in detrimental effects of protein nitration in the outflow pathway. As a result, these events likely contribute to tissue damage, altered protein function, and ultimately elevated IOP.^45^ However, the enhanced nitration we observed in endo-KO was more modest than that previously described in global KO. As mentioned elsewhere in this article, global Cav1 KO mice exhibited increased pulmonary hypertension; however, this pathology was absent in double eNOS and Cav1 KO mice. Hence, chronic hyperactivation of eNOS may be a secondary effect of Cav1 KO that produces pulmonary precapillary structural abnormalities that lead to pulmonary hypertension.^95^ Additionally, Zhao et al.^95^ (2009) determined that pulmonary hypertension in Cav1 KO mice was attributable to impairment of protein kinase G (PKG) activity via nitration at Tyr345 and Tyr549. Thus, the elevation of IOP in endothelial and global Cav1 KO mice might be partly due to CO tissue damage and dysfunctional PKG signaling after long-term exposure to peroxynitrite. This theory would be consistent with work that showed elevated levels of nitrotyrosine in the TM of human patients with POAG.^96^ Our data on tyrosine nitration in Cav1^ΔEC^ mice did not specify which proteins were affected. Thus, future work might involve testing the effects of blocking peroxynitrite formation and overexpressing PKG in endothelial and global Cav1 KO mice to determine whether nitrative stress induces PKG-dependent defects to the CO pathway. Moreover, the minimally increased tyrosine nitration in anterior segments of Cav1^ΔEC^ mice could be due to contamination from non-SC cells that still expressed Cav1, so future work might also involve performing immunostaining to see specific tissues with elevated tyrosine nitration. These future tests might aid in understanding the link between polymorphisms at the Cav1/Cav2 locus and the increased risk of POAG in humans.

It is important to note that transgenic mice overexpressing eNOS exhibit decreased the IOP and increased outflow facility that is attributable to an increase in NO signalling.^16^ If that increased IOP in endothelial and global Cav1 KO mice is at least partly attributable to tyrosine nitration, then this result would suggest innocuous levels of tyrosine nitration in eNOS-overexpressing mice, despite an increased production of NO. The lack of apparent CO dysfunction in eNOS-overexpressing mice might be due to negative regulation of eNOS by Cav1, and subsequent maintenance of NO at nontoxic levels. Moreover, as Cav1 is likely to be involved in mechanotransduction,^43^ the absence of Cav1 in global and Cav1^ΔEC^ mice might impair the ability of the CO tissue to detect and respond to changes in pressure and flow, leading to increased IOP.

**Figure 8.**
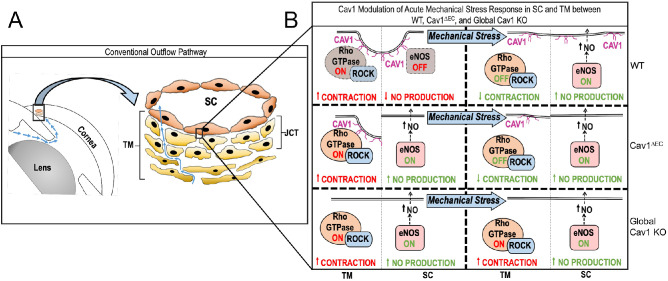
Schematic illustrating the physiologic response to mechanical stress on SC and TM in WT, global Cav1 KO, and Cav1^ΔEC^. (**A**) Illustration of the CO pathway. (*Left*) Low magnification image depicting lens, cornea, iridocorneal angle and direction of aqueous humor flow (*blue arrows*). (*Right*) Higher magnification illustration depicting SC, TM, and juxtacanalicular region with direction of aqueous humor flow (*blue arrows*). (**B**) Flow diagram demonstrating the status of Rho GTPase (in TM) and eNOS activity (in SC) before and after mechanical stress for WT, Cav1^ΔEC^, and global Cav1 KO. JCT, juxtacanalicular region.

Interestingly, outflow facility was impacted differently between global and endothelial Cav1 KO mice, as compared with respective WT mice. In global Cav1 KO mice, outflow facility was significantly decreased compared with WT mice,^43^ but outflow facility was unchanged between endothelial Cav1 KO mice and respective WT mice. Unlike in global Cav1 KO mice, Cav1 is still expressed in the TM of Cav1^ΔEC^ mice. Therefore, increased outflow resistance in global Cav1 KO mice is likely due to TM dysfunction, as illustrated in Figure 8. Cav1 and Cav2 expression in the TM have been implicated as crucial regulators of IOP and outflow facility.^91^ For example, increased activity of Rho/ROCK signaling is associated with reduced outflow facility and elevated IOP,^97^^,^^98^ and treatment with Rho kinase inhibitors effectively reduce IOP in patients with glaucoma.^99^^,^^100^ Transient knockdown of Cav1 in the TM potentiates downstream effectors of the Rho/ROCK signaling pathway, such as increased α-SMA expression and stress fiber formation,^91^ which might impede pressure-dependent regulation of IOP and outflow facility. Furthermore, the extracellular matrix is a crucial source of resistance in the CO pathway,^101^^,^^102^ and transient knockdown of Cav1 resulted in increased activity of matrix metalloproteinases.^91^ Thus, loss of Cav1 in the TM could affect normal regulation of IOP via elevated Rho/ROCK signaling or cause dysregulated extracellular matrix remodeling; however, these hypotheses need further investigation.

Another factor that might contribute to variations in outflow between endo- and global Cav1 KO mice is the expression of Cav2 in the TM. Transient Cav2 knockdown has been shown to decrease outflow facility in perfused post mortem porcine and human anterior segments.^91^ Because Cav2 deficiency is concomitant with Cav1 KO,^43^^,^^103^ it is possible that the remaining Cav2 expression in the TM of the Cav1^ΔEC^ mice enabled maintenance of IOP-regulating functions within the CO pathway. The role of Cav2 as a signaling modulator is unclear. Hence, more work is needed to investigate how Cav2 plays a role in the regulation of IOP and aqueous humor drainage.

When framing differences between outflow facility data from global and Cav1^ΔEC^ mice, it is important to consider that facility measurements are performed in enucleated eyes, whereas IOP is measured in living animals. Thus, measuring outflow facility ex vivo does not entirely capture the mechanisms that might influence outflow facility regulation in vivo.^64^ For example, there is no episcleral venous pressure in enucleated eyes, so the influence of vasomotor regulation on outflow facility will not be detected. Eliminating the effect of the episcleral venous pressure could at least partially mask the effect of endothelial Cav1 deficiency on outflow facility.

In conclusion, we have provided strong evidence that tissue-specific expression of Cav1, a POAG-associated gene product, is required for disbursement of NO and its downstream effect on IOP and outflow. We have demonstrated that elevated IOP in endothelial Cav1-KO mice is at least partly attributable to loss of caveolae, Cav1, or Cav2 from the cells of the SC and distal vessels. However, outflow facility defects were rescued by Cav1 expression in the TM of Cav1^ΔEC^ mice. We have also shown that eNOS hyperactivation, secondary to endothelial Cav1 KO, partially compensates for CO defects resulting from Cav1 deficiency. Thus, our data support the hypothesis that caveolae in SC and distal vessels regulate IOP and pressure-dependent CO via eNOS signaling. Moreover, we propose that defects in the CO pathway that lead to elevated IOP in global and Cav1^ΔEC^ mice might be partially due to chronically high NO levels leading to nitrative stress in the SC and distal vessels. More work is needed to determine the pathophysiology of CO defects resulting Cav1 (and caveolae) deficiency in the TM, but we propose that Cav1 modulates Rho/ROCK signaling in TM while controlling eNOS activity in the SC (Fig. 8). Regardless, results show that caveolae play a critical role in regulation of aqueous outflow and are a promising target for future investigations into therapeutic applications for POAG.

## Supplementary Material

Supplement 1
