# Supplementary material for: Physiologic Consequences of Caveolin-1 Ablation in Conventional Outflow Endothelia
Source: Invest Ophthalmol Vis Sci. 2020 Sep 17;61(11):32. doi: 10.1167/iovs.61.11.32 (PMC7500130; doi:10.1167/iovs.61.11.32)
Supplement: Supplement 1 [file iovs-61-11-32_s001.pdf]

Supplementary Information for:

Physiological consequences of caveolin-1 ablation in conventional outflow endothelia

Michael L. De Ieso[1], Jami M. Gurley[2], Mark E. McClellan[2], Xiaowu Gu[2], Iris Navarro[1], Guorong Li[1], Maria Gomez-Caraballo[1], Eric Enyong[3], W. Daniel Stamer[1*], and Michael H. Elliott[2,3 *]

[1] Department of Ophthalmology/Duke Eye Center, Duke University, Durham, 27710, NC, USA.

[2] Department of Ophthalmology/Dean McGee Eye Institute University of Oklahoma Health Sciences Center, Oklahoma City, 73104, OK, USA.

[3]Department of Physiology, University of Oklahoma Health Sciences Center, Oklahoma City, 73104, OK, USA.

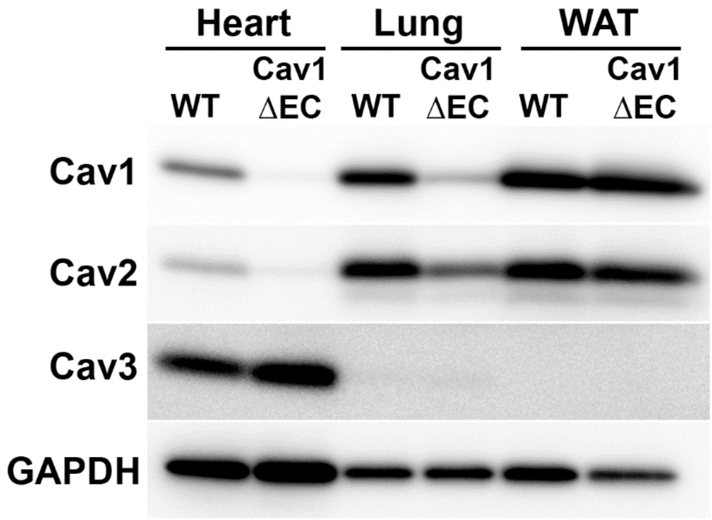

Supplementary Figure S1: Western blot depicting Cav1, Cav2, and Cav3 expression in different tissues of Cav1$^{\Delta EC}$ and WT mice. Dramatic reduction in total Cav-1 expression in Cav1$^{\Delta EC}$ mouse heart and lung tissues. Cav1 deletion in Cav1$^{\Delta EC}$ is not observed in white adipose tissue (WAT) which abundantly expresses Cav1 in adipocytes which are not targeted by Tie2-cre. Cav2, which requires Cav1 for stability, is also reduced in tissues in which Cav1 is deleted. As anticipated, Cav-3 expression in the heart was unaffected by endothelial Cav1 depletion as its expression in striated muscle does not depend on Cav1. Glyceraldehyde 3-phosphate dehydrogenase (GAPDH) was used as a reference housekeeping protein for protein normalization.

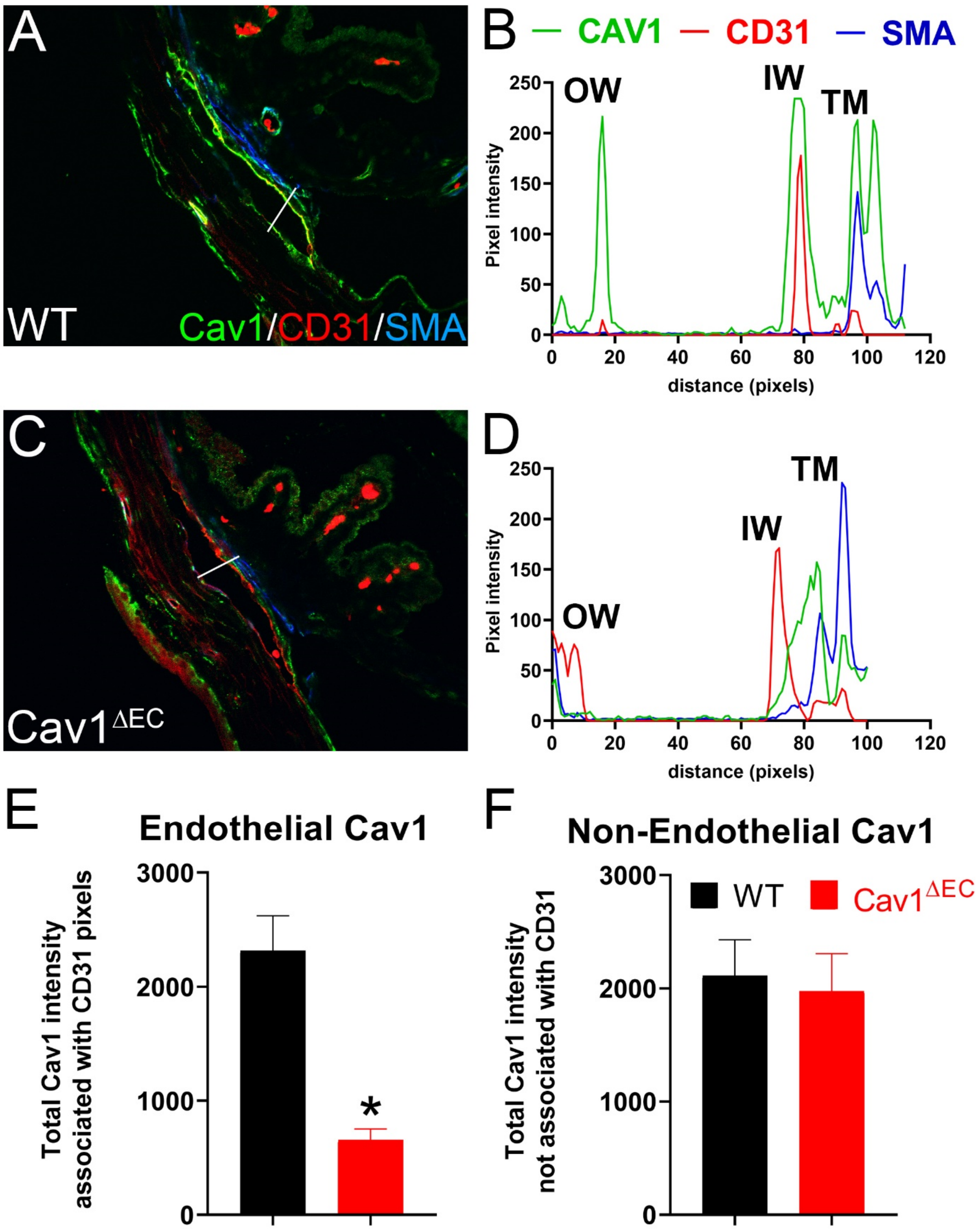

Supplementary FigureS2: Cav1 deletion efficiency and specificity determined by immunohistochemistry and density profile mapping. Representative confocal slices from WT (A) and Cav1$^{\Delta EC}$ (C) mice are shown with representative lines drawn across the SC lumen. The pixel intensities for each color channel along these lines are depicted in representative graphs for WT (B) and Cav1$^{\Delta EC}$ (D). The positions of the inner (IW) and outer (OW) walls of the SC and the

TM are indicated. A subset of TM is αSMA-immunoreactive and is indicated in blue. "Endothelial Cav1" was determined by assessing the total Cav1 intensity in pixels that were also positive for CD31 (E) and the "Non-Endothelial Cav1" (F) by assessing total Cav1 intensity in CD31-negative pixels. As indicated, a significant, ~72% reduction Cav1 signal intensity was observed in CD31-positive pixels whereas no significant reduction was observed in non-endothelial pixels ($p < 0.05$, unpaired t-test, n = 2 WT and 3 $Cav1^{\Delta EC}$).

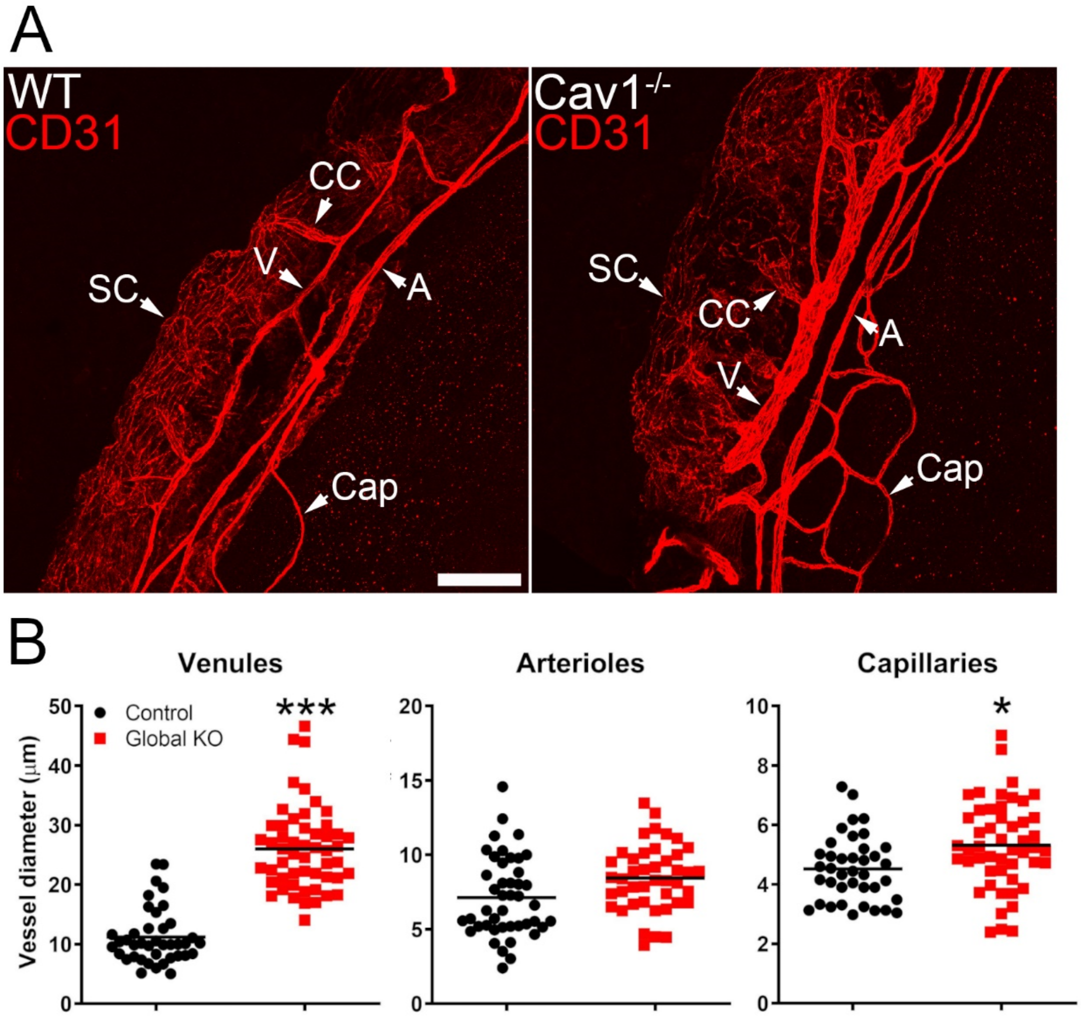

Supplementary Figure S3. Distal vessels are enlarged in global Cav1 KO mice. (A) Confocal microscopic projections of limbal tissues from WT and global Cav1 KO mice. Whole mounts were labeled with an endothelial marker, CD31 (red), which depicts distal vessels and capillaries. Global Cav1 KO mice showed enlarged distal vessels as compared to WT. Abbreviations: SC, Schlemm's canal; CC, collector channel; A, arteriole; Cap, capillary; V, venule. (B) Analysis of distal vessel diameters in venules, arterioles, and capillaries. Each point represents a single measurement along the vessel. Vessel diameter of distal arterioles was not significantly different between WT (n=5 mice) and global Cav1 KO (n=6 mice). Vessel diameter of was significantly increased in global Cav1 KO (n=6 mice) compared to WT (n=5 mice) for distal venules (***p = 0.0004) and distal capillaries (*p = 0.04). For all graphs, each point represents a single measurement along the vessel. For statistical analysis, each n-value is calculated as the mean of all measurements along a vessel for each mouse. All data analyzed using unpaired t-test. Scale bar, 100 μm.

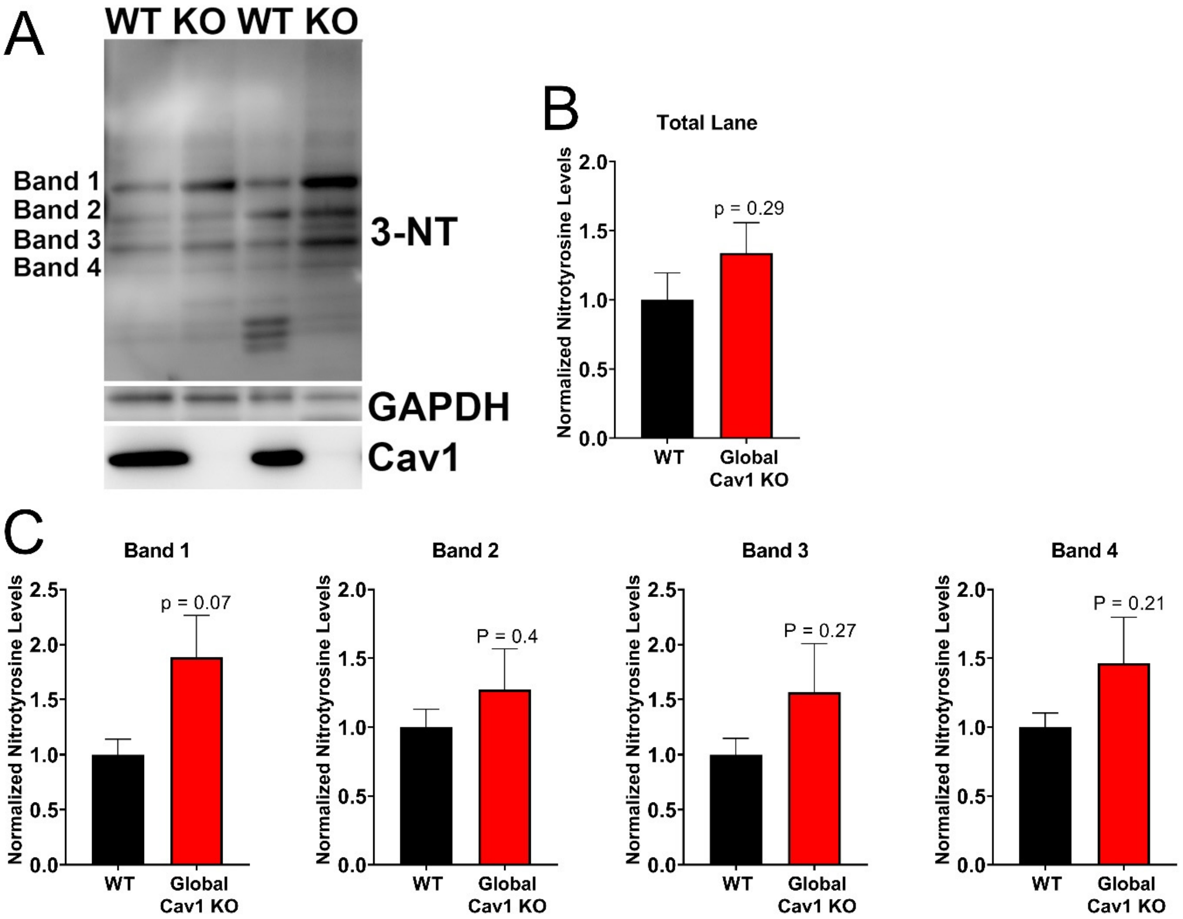

Supplementary Figure S4: Densitometric studies of nitrotyrosine levels in global Cav1 KO mice. (A) Immunoblots staining for nitrotyrosine (3-NT) in protein lysates extracted from iridocorneal angles of WT and global Cav1 KO mice. Each column represents protein extracted from a single mouse. There are four main bands evident. All signal intensities were normalized to GAPDH. Cav1 is completely absent in global KO mice. (B) Densitometric analysis depicting combined data for all four bands within each lane, for each group. When calculating total nitrotyrosine levels, there was no significant difference between WT and global Cav1 KO. All data analyzed using unpaired t-test, n=4 for all groups. All densitometry data were normalized to WT control. (C) Densitometric analysis depicting data for each separate band within each sample. There was an increase in tyrosine nitration of the global Cav1 KO compared to the WT samples for all bands but this was not significant.

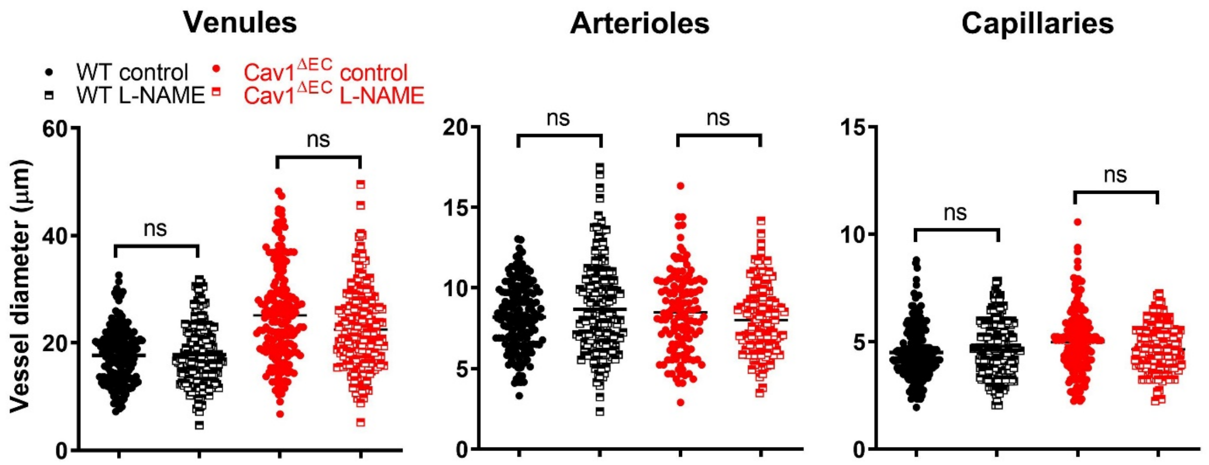

Supplementary Figure S5. Distal vessels size after L-NAME treatment in Cav1^{ΔEC} mice. Analysis of distal vein, artery, and capillary diameter after treatment with L-NAME. No significant reversal of distal vessel enlargement was observable in any vessel of the WT or Cav1^{ΔEC} mice (One-way ANOVA, Tukey post-hoc test, n=20-21). Each point represents a single measurement along the vessel. For statistical analysis, multiple vessel regions were used and counted as n-values for each mouse.
